# Neuromuscular adaptations to experimentally induced pain in the lumbar region: protocol for a systematic review and meta-analysis

**DOI:** 10.1186/s13643-021-01831-1

**Published:** 2021-10-15

**Authors:** Valter Devecchi, Deborah Falla, Hélio V. Cabral, Alessio Gallina

**Affiliations:** Centre of Precision Rehabilitation for Spinal Pain (CPR Spine), School of Sport, Exercise and Rehabilitation Sciences, College of Life and Environmental Sciences, University of Birmingham, Edgbaston, Birmingham, B15 2TT UK

**Keywords:** Back pain, Rehabilitation medicine, Neurophysiology

## Abstract

**Background:**

Numerous studies report changes in neuromuscular control in people with low back pain (LBP). However, the relationship between pain and altered neuromuscular control is challenging to unravel given the heterogeneity that exists in clinical populations. One approach commonly adopted to overcome this issue is the use of experimental pain models, but it is currently unclear if the effects of experimental pain are consistent between studies. Therefore, this planned study will systematically evaluate and summarise the effect of experimentally induced pain in the lumbar region on neuromuscular control at sites both locally and remote to the low back.

**Methods:**

This protocol has been developed following the Preferred Reporting Items for Systematic Review and Meta-Analysis Protocols (PRISMA-P). MEDLINE, EMBASE, CINAHL, ZETOC, Web of Science, and grey literature will be searched up to August 31, 2021. Screening processes (title/abstract and full-text), data extraction, and risk of bias assessment will be conducted by two independent reviewers. Studies investigating the effects of exogenous pain models delivered to the low back region on neuromuscular control in healthy individuals will be included. Muscle activity and body kinematics will be the outcomes of interest. The comparisons of interest will be between baseline or control conditions and the experimental pain condition, as well as between the experimental pain and post-pain conditions. Randomised crossover and non-randomised studies of interventions will be included and their risk of bias will be evaluated with the Cochrane Risk-of-Bias tool or with the Risk Of Bias In Non-randomised Studies of Interventions tool, respectively. A random-effect meta-analysis will be conducted for quantitative synthesis when clinical and methodological consistency is ensured. Quality of evidence will be evaluated using the Grading of Recommendations, Assessment, Development and Evaluation guidelines.

**Discussion:**

The current review will provide new insights to understand if and what neuromuscular adaptations are caused by pain experimentally induced in the lumbar region. Our findings will reveal which experimental pain model is able to better reproduce adaptations similar to those identified in people with low back pain, possibly contributing to improving our understanding of motor adaptation to low back pain in the long term.

**Systematic review registration:**

PROSPERO CRD42020220130

**Supplementary Information:**

The online version contains supplementary material available at 10.1186/s13643-021-01831-1.

## Background

Pain is an essential component of our life because it acts as an alarm system to ensure protection. However, pain can persist despite the absence of noxious stimuli, leading to the development of a chronic condition which becomes pathological rather than physiological [1]. Although the proposal of a biopsychosocial model of pain helped to disentangle the multidimensional nature of pain and the transition from acute to chronic, the understanding and treatment of pain still remains one of the biggest challenges for our society—so much so that low back and neck pain remain leading causes of disability worldwide [2].

Besides the role of psychosocial aspects in persistent pain [3, 4], clinical evidence has revealed extensive changes in neuromuscular control in people with pain, during both acute and chronic stages [5–9]. Current theories suggest a strong relationship between pain and movement, implicating changes in response to pain at multiple levels of the neuromuscular system [1, 10, 11]. However, the causal link between pain and neuromuscular control is challenging to unravel within a clinical population, given the co-existence of other factors and the clinical heterogeneity that exists in people with musculoskeletal disorders. The use of observational designs is affected by the bias introduced by potential confounders, such as the subjective report of pain and the interplay between physical and negative cognitive/emotional factors (e.g. fear, anxiety, depression, pain catastrophizing) [12]. Additionally, the pre-existence of altered neuromuscular control cannot be ruled out in cross sectional studies.

In order to overcome these limitations, the use of experimental pain models in healthy individuals represents an appealing approach to better standardise and investigate the effects of nociception on the neuromuscular system [13–15]. Moreover, interventional studies allow to define what variable acts as cause or effect in the investigated relationship and to collect reference values of neuromuscular control at the baseline. Experimental pain models using mechanical, thermal, chemical, or electrical stimuli can target different anatomical tissues and nociceptors [13–15]. Endogenous stimuli aim to recreate the experience of pain though physiological conditions (e.g. exercise or ischemia), while exogenous models achieve the same purpose through external sources [13, 16]. Different experimental pain models can also be used to modulate the duration of pain experienced. For example, hypertonic saline solution, electrical stimulation, and nerve growth factor are able to reproduce tonic, phasic, and sustained pain, respectively [15, 16]. Thus, when the aim is to understand whether pain changes neuromuscular control, experimental pain models may help to overcome some of the limitations encountered in the investigation of neuromuscular control in clinical populations.

A summary of the evidence regarding the effects of experimentally induced pain in the lumbar region can provide a broad overview of the influence of pain on neuromuscular control, both locally and remotely. Although the consequences of experimental limb pain have been detailed in a previous systematic review [17], findings cannot be directly translated to the lumbar region given the biomechanical and sensorimotor complexity of the spine. Therefore, this systematic review aims to investigate whether neuromuscular control changes are observed when pain is experimentally induced in the low back region.

## Methods/design

### Primary review question

1. Does experimentally induced pain in the lumbar region induce neuromuscular adaptations in healthy adults?

### Secondary review questions

2.a- Are neuromuscular adaptations induced both locally and remote to the lumbar region?
2.b- Do neuromuscular adaptations outlast the duration of the painful stimulus?
2.c- Do neuromuscular adaptations depend on the type of experimental pain model?

This protocol has been developed following the Preferred Reporting Items for Systematic Review and Meta-Analysis Protocols (PRISMA-P, see Additional file 1) [18] and the second edition of the Cochrane Handbook for Systematic Reviews of Interventions [19]. A prospective registration of the present protocol has been conducted on PROSPERO [CRD42020220130]. Results from the present systematic review and meta-analysis will be reported following the PRISMA 2020 statement [20].

### Eligibility criteria

The inclusion and exclusion criteria of the studies to be included in the review are detailed using the PICOS (P: Population; I: Intervention; C: Comparator; O: Outcome(s); S: Study design) framework [18]. A list of items that will be considered for each element of the PICOS framework is presented below (Data items and outcome prioritisation).

### Population

Healthy adults (> 18 years) represent the population of interest for this systematic review. Studies that include only participants with current or a history of musculoskeletal disorders will be excluded.

### Interventions

Studies investigating neuromuscular adaptations to experimentally induced pain in the lumbar region will be included. Because of their ability to specifically induce pain in a target body region, only exogenous pain models consisting of the application of electrical, thermal, or chemical stimuli will be considered. Studies where pain was induced by endogenous models such as eccentric exercise will be excluded since this would introduce additional factors (presence of muscle fibre damage, fatigue etc.), which may also influence neuromuscular control. Studies that induced pain with prolonged standing protocols will be excluded since they do not specifically target the lumbar region. Table 1 presents a summary of the experimental pain models which will be included in the review [13, 15].

**Table 1:** Experimental pain models eligible as interventions

Injection/topical application of algesic substances	
■ Hypertonic saline ■ Capsaicin ■ Glutamate ■ Acidic buffers ■ Miscellaneous (e.g. serotonin, bradykinin, histamine) ■ Nerve growth factor	
Thermal stimulation	
■ Cold ■ Contact heat ■ Laser	
Electrical nociceptive stimulation	

If participants received more than one experimental pain model at the same time (e.g. hypertonic saline injection and delayed onset muscle soreness), without evaluating the effects of the intervention of interest when delivered individually, the study will be excluded.

### Comparators

To simplify the description of the comparisons of interest, four time points in the evaluation of neuromuscular control will be considered.

- Baseline (BASE): assessment conducted before any interventions are delivered.
- Control (CTR): assessment conducted when a control intervention is delivered (e.g. isotonic saline).
- Experimentally induced pain (PAIN): assessment conducted when participants are experiencing pain induced by the intervention of interest.
- Post-pain (POST): assessment conducted when, after the induction of pain, the experience of symptoms is resolved (or only minimal pain is reported, see below).

Only assessments conducted on the same participant during different conditions will be considered eligible for comparison (within-subject design). Based on the time points reported and the review questions, different comparisons of interest are identified. If a study did not test all four time points (e.g. did not include a control condition or did not test participants after pain has resolved), the study will be included and only the time points tested will be considered.

In order to address the primary review question (1), neuromuscular control evaluated at baseline and after a control intervention (BASE and CTR) will be compared with assessment of neuromuscular control during the pain condition (PAIN). Data from the baseline assessment needs to be included because a control intervention cannot be delivered for all experimental pain models. Consequently, a subgroup analysis investigating the effects of different nociceptive stimuli on neuromuscular control (review question 2.c) can only be conducted using data from the baseline assessment. On the other hand, data from control interventions provides higher quality evidence being less affected by potential confounders. For these reasons, both comparisons will be included.

The secondary review question 2.b will be addressed when a study includes the evaluation of neuromuscular control after the resolution of symptoms (during the same session). Thus, the POST condition will be compared with the PAIN condition. The secondary review question 2.c will be explored through a subgroup analysis comparing the BASE and PAIN conditions based on the experimental pain model adopted.

### Outcomes

The assessment of changes in neuromuscular control is the focus of this review. Therefore, the evaluation of body kinematics and muscle activity are the broad outcome domains of interest. Studies where voluntary or automatic (e.g. postural) tasks were assessed will be included. Other outcome domains related to neuromuscular control during experimentally induced pain (force and corticospinal excitability of muscles) will not be included because they are already investigated in other reviews, currently registered on PROSPERO (CRD42020196479 and CRD42018095693).

The evaluation of muscle activity will include the assessment of the intensity, recruitment, and onset of muscle activation. Electromyography (intramuscular and surface), ultrasound, and muscular functional MRI are the measurement tools considered.

Range of motion, speed, quality, and variability of movements will be the narrow outcome domains of interest for body kinematics. The measurement tools considered are motion analysis systems, such as optoelectronic systems and inertial measurement units (IMUs).

No limitations will be set regarding the body region investigated in order to address the secondary review question 2.a. This approach will allow us to understand if experimental pain induced in the low back induces adaptation of muscle activation and kinematics locally in the lumbar region and at remote sites.

### Study designs

Randomised trials (crossover design only) and non-randomised studies of interventions (NRSI, repeated measures design) will be included. NRSI will be included since, considering the scoping search and the algorithm described in the Handbook for Systematic Reviews of Interventions [21], there are insufficient randomised crossover trials addressing the PICO. Furthermore, a control intervention necessary for randomisation is not available for all experimental pain models.

### Language

Studies will be included when reported in English, Italian or Portuguese. Articles with relevant titles and abstracts reported in other languages will be excluded and listed in an appendix.

### Information sources

The search will be conducted by one reviewer (VD) from inception until the 31 of August 2021. The following databases will be searched: MEDLINE (OVID interface), EMBASE (OVID interface), CINAHL (EBSCO interface), and ZETOC. Moreover, specific Internet sites will be searched as well, including PubMed and Web of Science (Clarivate Analytics). Hand-searching will be conducted based on the results of the scoping search and considering journals relevant for this review topic; specifically, *PAIN*, *Journal of Neurophysiology*, *Journal of Pain*, *European Journal of Pain*, and *Musculoskeletal Science and Practice*. In addition to this, reference lists of included studies and relevant reviews will be checked.

In order to reduce the risk of publication bias, grey literature databases will be searched (OpenGrey and Ethos), as well as dissertation abstracts and conference proceedings (*International Association for the study of pain World Congress*, *International Society of Electrophysiology and Kinesiology Congress*, *World Congress of Biomechanics*, *International Society of Biomechanics Congress*). Moreover, relevant authors in the field will be contacted to collect information about unpublished data or ongoing projects.

### Search strategy

Date, region, and language will not represent elements of restriction for the search. The search strategy, as well as the search process, will be developed and conducted by one reviewer (VD) with the support of an experienced librarian. To ensure a high sensitivity of the search, interventions and body region stimulated are the concepts considered into the search strategy, and they will be connected as follows:

(“experimental pain” OR “pain model”) AND (“back pain” OR “low back”)

where “experimental pain” includes all free-text words commonly adopted to report the use of experimental pain in a study (e.g. “experimental pain”, “pain induced”, “induced pain”, etc.) and “pain model” identifies the interventions (e.g. hypertonic saline, capsaicin, glutamate, electrical stimulation, etc.). Terms referring to the same concepts will be separated by the Boolean operator “OR”. Proximity searching will be used when possible. An example of search strategy on MEDLINE (OVID interface) is reported in Table 2. The search will be conducted using subject headings (MeSH) in addition to free-text. The search strategy will be adapted across different databases, but consistencies of searches will be ensured (a comprehensive reporting of the search for all databases is presented in Additional file 2).

**Table 2:** Search strategy on MEDLINE (OVID interface)

1. Experimental pain	((Experiment* adj5 pain) or (experimentally-induced adj4 pain) or (pain-induced or “experimental induced” or “experimentally induced”) or (induced adj3 pain) or “induced LBP” or (noxious adj3 stimul*) or (nociceptive adj3 stimul*) or (pain* adj3 stimul*)).mp
2. Pain model	(((“hypertonic saline” or capsaicin or glutamate or “laser evoked potential” or “laser evoked potentials” or “nerve growth factor”) and pain) or (electric* adj2 pain*) or (electric* adj2 stimul*) or (mechanic* adj2 pain*) or (mechanic* adj2 stimul*) or (thermal* adj2 pain*) or (thermal* adj2 stimul*) or (chemical* adj2 pain*) or (chemical* adj2 stimul*) or (cutaneous adj2 pain*) or (cutaneous adj2 stimul*)).mp or Saline Solution, Hypertonic/ or Electric Stimulation/
3. Body region stimulated	Back Pain/ or (“back pain” or “back ache” or backache*).mp or exp Low Back Pain/ or (“low back pain” or “lower back pain” or lumbago or LBP or “lumbar pain” or “lumbar spine” or “low back” or “lower back”).mp
4.	1 OR 2
5.	3 AND 4

### Data management

The management of data, inclusion of citations, abstracts, and full-text of relevant studies will be conducted using EndNote V.X9 (Clarivate Analytics). Studies will be uploaded during the searching process and duplicate removed by one reviewer (VD). When the list of searched studies is completed, it will be uploaded on Rayyan [22], a web-based application that will be used by two reviewers (VD and HC) to facilitate the screening process. Full text of the records that will be considered potentially eligible will be stored in EndNote V.X9, and their screening will be conducted on Rayyan, as well.

### Selection process

Every screening process will be conducted by two independent reviewers (VD and HC). In the first stage, titles and abstracts will be assessed against a piloted screening tool considering the eligibility criteria and the primary review question. Disagreement between reviewers will be resolved though discussion. If no consensus can be reached, a third reviewer (AG) will mediate the process. Then, full-text records will be retrieved for potentially eligible studies, and, in a second stage, their screening will be conducted. Again, a third reviewer (AG) will be consulted for arbitration. During both stages of the screening process, the agreement between the two reviewers will be assessed using the kappa statistic. The PRISMA flow diagram will used to summarise the study selection process [18].

### Data extraction process

Data extraction will be performed by one reviewer (VD), followed by a verification of accuracy conducted by a second reviewer (HC). At the beginning, three reviewers (VD, HC, AG) will pilot a data extraction form on four included studies. The form will be developed on Microsoft Excel. A description of data items and outcomes that will be extracted is provided in the following section and summarised in Table 2. Disagreement between reviewers will be resolved by discussion. Where necessary, a third reviewer (AG) will be consulted to mediate. When data related to the outcome of interest are not reported in a usable format for data synthesis, they will be extracted from figures with WebPlotDigitizer, a software that allows to digitalise data points in an image and extract the numerical information of interest. This plot digitising tool has high intercoder reliability and validity when extracted data was compared with the original data [23]. Authors of primary studies will be contacted when the methods and results are ambiguous or unpublished information are necessary for data synthesis. Authors will be contacted no more than twice; if a reply is not obtained after an initial email, a second one will be sent after fifteen days. If a reply to the reminder email is not obtained, data will be considered irretrievable. When multiple records of the same study are identified, they will be collated and the one with the most comprehensive description and data reporting will be considered as the best source for data synthesis [24].

### Data items and outcome prioritisation

The variables of interest are selected considering the PICOS framework, as well as the information necessary to support the risk of bias (RoB) assessment and data synthesis. Therefore, details regarding report identification features, sample characteristics, interventions delivered, comparators, outcomes of interest, and study methods/design will be extracted. A completed list of these variables is reported in Table 3. After their extraction, this information will be reported in the table “Characteristics of included studies”.

**Table 3:** Characteristics that will be extracted from included studies

Identification features of the report	Authors Title Year Source (e.g. journal article, conference abstract)
Population	Sample size Age Gender Height, weight, body mass index Randomisation details and arm group characteristics (crossover design only)
Intervention	Experimental pain model/s adopted Intervention characteristics (e.g. type, dosage, method) Body region stimulated (anatomical structure and location, unilateral or bilateral stimulation) Average and highest level of pain experienced (VAS or NRS) Duration of pain symptoms Qualitative description of pain (e.g. McGill Pain Questionnaire) Perceived location and distribution of pain symptoms (including referred pain) Co-interventions Potential confounders to the intervention effect (NRSI only) Deviations from intended intervention Time window between interventions
Comparator	No intervention (baseline condition) or control intervention Typology of control intervention Level of pain induced with the control intervention (minimal versus not at all) Duration of pain symptoms (if experienced) Co-interventions Assessment of the POST-PAIN condition Time window between PAIN and POST-PAIN condition
Outcomes	Outcome domain Outcome measure Measurement tool Body region/muscle investigated (including all spinal regions and limbs) Task
Design	Randomised trial (crossover) or NRSI (repeated measure) Time point assessments, including their order and time in between (e.g. wash-out period)

Furthermore, information necessary for data synthesis involves the number of participants included in the analyses, summary data (means and standard deviation) for each condition investigated (BASE, CTR, PAIN, POST), and effect estimates between them.

In this systematic review, two broad outcome domains are identified: muscle activity and body kinematics. Therefore, the primary outcomes that will be reported in the table “summary of findings” are muscle activation intensity/recruitment, timing of muscle activation, and kinematics. Results obtained from the investigation of the lumbar region will be separated from those assessing appendicular regions and thoracic/cervical spine (to address the review question 2.a). The time points described previously (BASE, CTR, PAIN, POST) will be considered for the extraction of the outcomes of interest. Moreover, when an outcome of interest is evaluated at different time points after the resolution of pain, only the first pain-free time point will be included.

### Risk of bias

Two independent reviewers (VD and HC) will assess the RoB. Disagreement will be resolved by discussion, and, when necessary, a third reviewer (AG) will be consulted for arbitration. Based on the study designs included in this review, two RoB tools will be used. The second version of the Cochrane risk-of-bias tool (RoB2) [25] will be used for crossover trials [26]. Specifically, bias arising from the randomisation process, period and carryover effects, deviations from intended interventions, missing outcome data, measurement of the outcome, and selection of reported results will be assessed [27]. Findings from repeated measure assessments will be evaluated with the ROBINS-I tool for uncontrolled before-after designs [21, 27]. Therefore, bias arising from selection of participants, classification of interventions, deviation from intended interventions, missing data, measurement of outcomes, and selection of the reported result will be evaluated [27]. These tools have been selected because they involve a similar structure and evaluation process. Moreover, for both tools, all items will receive the same weight. From a methodological perspective, both tools adopt a domain-based approach that is preferred to checklists or scales that lead to a summary of multiple components into a single number. Overall, the RoB2 for crossover trial identifies three potential risk-of-bias judgments: low risk of bias, some concerns, and high risk of bias. The ROBIN-I defines a risk-of-bias judgement as low, moderate, serious, and critical (each tool provides specific interpretations). If a meta-analysis will be conducted, overall risk of bias will be presented for each individual study.

In addition to the source of information to support the RoB judgement, a graphical representation developed with R software (version 4.1.0) [28] will be provided to summarise the RoB in each domain. Adopting the process described previously, authors of a study will be contacted no more than twice to clarify ambiguous information. If a reply is not obtained, the RoB for that specific domain will be reported as ‘unclear’.

The RoB identified will not preclude findings of a study to be included in the data synthesis. Therefore, results from all included studies will be presented and a narrative discussion of the RoB will be reported. Furthermore, the assessment of the RoB will be used to summarise the quality of evidence for each outcome domain (see the “Confidence in cumulative estimate” section) [29].

### Data synthesis

Information from all variables described in the previous section will be collected and grouped in a customised table to manage the synthesis of findings. Quantitative syntheses will be considered for the primary and secondary review questions (1 and 2.b) and, because of the designs of included studies, effect estimates will be computed from within-subject change scores (i.e. mean change between conditions normalised by the standard deviation of the change). This approach will ensure methodological consistency and homogeneity in the use of summary statistics across studies. When all raw data cannot be obtained, effect estimates will be computed from the available information and following the guidelines described in the *Cochrane Handbook* [30].

#### Does experimentally induced pain in the lumbar region induce neuromuscular adaptations in healthy adults?

Initially, methodological consistency will be assessed to decide if findings of the included studies can be summarised quantitatively. Meta-analysis from two or more studies will be conducted when studies show homogeneity regarding the comparator condition and the outcome evaluated, in terms of domain and body region assessed (e.g. the effect of hypertonic saline injection in the lumbar erector spinae on lumbar erector spinae activity measured by EMG compared to baseline measures). Effect estimates from the comparisons between the PAIN and BASE conditions, and between the PAIN and CTR conditions will be extracted and reported in the “Main findings” Table. Data from baseline and a control intervention will not be pooled for synthesis because the latter provides a higher quality evidence controlling for potential confounders. However, a control intervention will not be available for all experimental pain models included.

The *I*^*2*^ statistic will be preferred to the Cochran’s *Q* test in the analysis of statistical heterogeneity because the latter has a low power when small studies are included in a meta-analysis [31]. Moreover, information regarding the amount of heterogeneity are more useful rather than a rigid cut-off value [32]. We anticipate that findings from the comparison between baseline and pain conditions could be affected by substantial heterogeneity (*I*^*2*^ > 50%); therefore, it will be explored with a pre-specified subgroup analysis taking into account the use of different experimental pain models. This subgroup analysis will allow us to explore the secondary question of this review (2.c; Do neuromuscular adaptations depend on the type of experimental pain model?). Considering the difficulty to obtain a large number of studies with a homogeneous methodology, results from subgroup analysis will likely be reported narratively and graphically with a forest plot [33].

Quantitative synthesis will be performed using a random-effect model with an inverse-variance method [30], as recommended by the Cochrane Back and Neck group [34]. The selection of this approach is influenced by the heterogeneity existing in how the nociceptive stimulus is delivered across different experimental pain models, and the inclusion of NRSI.

Effect estimate for continuous outcomes will be reported using the standardised mean difference, and its variance will be presented with 95% confidence interval (CI). Although rarely used to describe the outcomes of interest for this review, we anticipate that findings regarding dichotomous outcomes will be summarised using the risk ratio (with 95% CI). When effect estimates are adjusted for important confounders that can impact findings, they will be extracted from the analysis conducted by the study authors and variables considered as confounders will be reported.

#### Do neuromuscular adaptations outlast the duration of the painful stimulus?

The same criteria of between-study homogeneity described previously will be adopted, with quantitative synthesis. However, instead of baseline and control intervention, the POST pain condition will be used as comparator with the PAIN condition. Again, random-effect model with inverse-variance method will be adopted and statistical heterogeneity will be assessed with the *I*^*2*^ statistic [30]. A subgroup analysis will be performed to explore the heterogeneity introduced by different pain models. Similarly, RoB of individual studies will be considered to explain potential heterogeneity across findings.

Quantitative data synthesis will be graphically presented with forest plots along with the overall RoB for each individual study. Quantitative data synthesis and forest plots will be developed using R software [28]. In addition to meta-analyses, a systematic narrative synthesis and a structured tabulation will be provided for all results [33, 35]. Findings from studies will be grouped based on the comparison conducted, the outcome domain investigated, and the experimental pain model adopted. A graphical description using a forest plot will support the vote-counting synthesis regarding the direction of effect for different comparisons, as well as the identification of potential source for heterogeneity [33]. Risk of bias will not represent elements of restriction in the presentation of findings.

### Meta-biases

Initially, the risk of publication bias will be addressed searching grey literature databases, dissertation abstracts, and conference proceedings (see the “Information sources” section) [36]. Multiple information from the same study will be grouped to analyse if discrepancy exists between the planned analysis and reported findings. When results that should be included are completely unavailable, study authors will be contacted.

### Confidence in cumulative estimate

Certainty of the body of evidence will be assessed using the Grading of Recommendations Assessment, Development, and Evaluation (GRADE) approach and its associated guidelines [37, 38]. Quality of evidence will be reported for comparisons addressing the review questions (1 and 2.b). Furthermore, certainty of evidence will be analysed for each outcome domain included in the PICOS (muscle amplitude/recruitment, muscle timing, and kinematics), and it will be reported in the “Summary of findings” table [39]. For each important outcome domain, the table will include information regarding the population (overall number of participants and included studies), experimental pain model used, comparison, body region investigated (spine vs limbs), GRADE assessment for each domain, and overall certainty of evidence. Explanatory reasons in the judgement of the quality of evidence will be provided as well.

Overall, four levels of evidence are identified through the GRADE approach: ‘High’, ‘Moderate’, ‘Low’ and ‘Very Low’ [40]. Although non-randomised studies of interventions will be included, all findings will start as high certainty of evidence because their RoB will be assessed using the ROBINS-I [39]. After the initial level of certainty is established, it could be downgraded by one or two levels based on the assessment of five domains: study limitations, inconsistency, indirectness, imprecision, and publication bias. Instead, large effect estimate, dose response gradient and potential confounding that can underestimate the effect estimate, could upgrade the level of evidence [39, 41]. Risk of bias limitations will be obtained from the use of the appropriate tool (RoB2 or ROBIN-I). Pre-specified subgroup analysis (use of different experimental pain model and RoB) will be used to explore potential source of heterogeneity and, when it remains unexplained, the inconsistency will affect the certainty of evidence.

## Discussion

Findings from this systematic review will reveal if and what adaptations of the neuromuscular system are caused by pain induced in the lumbar region. These adaptations may be relevant to explain motor adaptation in clinical populations. For example, adaptations developed during experimental pain could explain some of the mechanisms of the transition from acute to chronic pain, or help identify effect modifiers or prognostic factors relevant for the prescription of treatments for people with low back pain.

Regarding the transition from acute to chronic pain, initial evidence will be provided by this review to understand whether nociception triggers only short-term adaptations (directly related to the experience of pain) or if persistent changes with potential long-term consequences could be involved.

Considering the neurophysiological characteristics of different pain models, it will be possible to understand if specific stimulations of the nociceptive system result in different neuromuscular adaptations. By showing which specific experimental pain model better replicates features of motor adaptation to pain observed in clinical low back pain, our findings will help researchers choose the most appropriate experimental methodology, ultimately resulting in experimental pain models that replicate clinical low back pain more closely. Moreover, findings could also represent a basis for new or combined experimental pain models.

## Supplementary Information

**Additional file 1.** PRISMA-P checklist.

**Additional file 2.** Search strategy used on all databases.

## Data Availability

Not applicable

## Abbreviations

BASE: Baseline condition
CTR: Control condition
PAIN: Experimentally induced pain condition
POST: Post-pain condition
NRSI: Non-randomised studies of interventions
RoB: Risk of bias
ROBINS-I: Risk Of Bias in Non-randomised Studies - of Interventions
GRADE: Grading of Recommendations Assessment, Development, and Evaluation
