# Supplementary material for: Neuromuscular adaptations to experimentally induced pain in the lumbar region: protocol for a systematic review and meta-analysis
Source: Syst Rev. 2021 Oct 15;10:270. doi: 10.1186/s13643-021-01831-1 (PMC8520254; doi:10.1186/s13643-021-01831-1)
Supplement: Supplementary file 2 — Additional file 2. Search strategy used on all databases. [file 13643_2021_1831_MOESM2_ESM.pdf]

SUPPLEMENTARY FILE 2

MEDLINE (OVID interface)

1. ((Experiment* adj5 pain) or (experimentally-induced adj4 pain) or (pain-induced or 'experimental induced' or 'experimentally induced') or (induced adj3 pain) or 'induced LBP' or (noxious adj3 stimul*) or (nociceptive adj3 stimul*) or (pain* adj3 stimul*)).mp

2. ((('hypertonic saline' or capsaicin or glutamate or 'laser evoked potential' or 'laser evoked potentials' or 'nerve growth factor') and pain) or (electric* adj2 pain*) or (electric* adj2 stimul*) or (mechanic* adj2 pain*) or (mechanic* adj2 stimul*) or (thermal* adj2 pain*) or (thermal* adj2 stimul*) or (chemical* adj2 pain*) or (chemical* adj2 stimul*) or (cutaneous adj2 pain*) or (cutaneous adj2 stimul*)).mp or Saline Solution, Hypertonic/ or Electric Stimulation/

3. Back Pain/ or ('back pain' or 'back ache' or backache*).mp. or exp Low Back Pain/ or ('low back pain' or 'lower back pain' or lumbago or LBP or 'lumbar pain' or 'lumbar spine' or 'low back' or 'lower back').mp

4. 1 or 2

5. 3 and 4

EMBASE (OVID interface)

1. ((Experiment* adj3 pain) or (experimentally-induced adj3 pain) or (pain-induced or 'experimental induced' or 'experimentally induced') or induced adj3 pain) or 'induced LBP' or (noxious adj3 stimul*) or (nociceptive adj3 stimul*) or (pain* adj3 stimul*)).mp

2. ((('hypertonic saline' or capsaicin or glutamate or 'laser evoked potential' or 'laser evoked potentials' or 'nerve growth factor') and pain) or (electric* adj1 pain*) or (electric* adj1 stimul*) or (mechanic* adj1 pain*) or (mechanic* adj1 stimul*) or (thermal* adj1 pain*) or (thermal* adj1 stimul*) or (chemical* adj1 pain*) or (chemical* adj1 stimul*) or (cutaneous adj1 pain*) or (cutaneous adj1 stimul*)).mp or Saline Solution, Hypertonic/ or Electric Stimulation/

3. Back Pain/ or ('back pain' or 'back ache' or backache*).mp. or exp Low Back Pain/ or ('low back pain' or 'lower back pain' or lumbago or LBP or 'lumbar pain' or 'lumbar spine' or 'low back' or 'lower back').mp

4. 1 or 2

5. 3 and 4

CINAHL (EBSCO interface)

((experiment* N5 pain) OR (experimentally-induced N4 pain) OR pain-induced OR “experimental induced” OR “experimentally induced” OR (induced N3 pain) OR “induced LBP” OR (noxious N3 stimul*) OR (nociceptive N3 stimul*) OR (pain* N3 stimul*) OR ((“hypertonic saline” OR capsaicin OR glutamate OR “laser evoked potential” OR “laser

evoked potentials” OR “nerve growth factor”) AND pain) OR (electric* N2 pain*) OR (electric* N2 stimul*) OR (mechanic* N2 pain*) OR (mechanic* N2 stimul*) OR (thermal* N2 pain*) OR (thermal* N2 stimul*) OR (chemical* N2 pain*) OR (chemical* N2 stimul*) OR (cutaneous N2 pain*) OR (cutaneous N2 stimul*)) [TX all text]

AND

(“back pain” OR “back ache” OR backache* OR “low back pain” OR “lower back pain” OR lumbago OR LBP OR “lumbar pain” OR “lumbar spine” OR “low back” OR “lower back” OR (MH “low back pain+”) OR (MH “back pain+”)) [TX all text]

ZETOC

1. experimental* AND “back pain”
2. “hypertonic saline” AND “back pain”
3. capsaicin AND “back pain”
4. electrical* AND “back pain”
5. thermal* AND “back pain”
6. chemical* AND “back pain”
7. cutaneous AND “back pain”
8. “nerve growth factor” AND “back pain”

PubMed

((“experimental pain”[tw] OR “experimentally-induced pain”[tw] OR “experimentally induced”[tw] OR “pain induced”[tw] OR “induced pain”[tw] OR “induced back pain”[tw] OR “induced low back pain”[tw] OR “experimental low back pain”[tw] OR “experimental back pain”[tw] OR “experimental muscle pain”[tw] OR “experimental LBP”[tw] OR “induced LBP”[tw] OR “noxious stimulation”[tw] OR “noxious stimuli”[tw] OR “nociceptive stimulation”[tw] OR “nociceptive stimuli”[tw] OR “noxious stimulus”[tw] OR “nociceptive stimulus”[tw] OR “pain stimulus”[tw] OR “pain stimulation”[tw] OR “pain stimuli”[tw] OR “painful stimulation”[tw] OR “painful stimuli”[tw] OR “painful stimulus”[tw])

OR

(“saline injection”[tw] OR “hypertonic saline”[tw] OR “buffered acidic”[tw] OR “acidic saline”[tw] OR capsaicin[tw] OR "capsaicin"[mesh] OR glutamate[tw] OR “laser evoked potential”[tw] OR “laser evoked potentials”[tw] OR “nerve growth factor”[tw] OR “electrical stimulation”[tw] OR “electrical pain”[tw] OR “electrical stimulus”[tw] OR “electrical stimuli”[tw] OR “thermal stimulation”[tw] OR “thermal stimuli”[tw] OR “thermal stimulus”[tw] OR “thermal pain”[tw] OR “chemical pain”[tw] OR “chemical stimulation”[tw] OR “chemical stimulus”[tw] OR “chemical stimuli”[tw] OR “cutaneous stimulation”[tw] OR “cutaneous stimuli”[tw] OR “cutaneous stimulus”[tw] OR “heat pain”[tw] OR "Saline Solution, Hypertonic"[mesh] OR "Electric Stimulation"[mesh]))

AND

(“back pain”[tw] OR “low back pain”[tw] OR backache[tw] OR backaches[tw] OR LBP[tw] OR lumbago[tw] OR “lumbar pain”[tw] OR “back ache”[tw] OR “lumbar spine”[tw] OR “back pain, low”[mesh])

WEB OF SCIENCE

TI/AB = ((experiment* NEAR/1 pain) OR (experimentally-induced NEAR/1 pain) OR pain-induced OR “experimental induced” OR “experimentally induced” OR (induced NEAR/1 pain) OR “induced LBP” OR (noxious NEAR/1 stimul*) OR (nociceptive NEAR/1 stimul*) OR (pain* NEAR/1 stimul*) OR ((“hypertonic saline” OR capsaicin OR glutamate OR “laser evoked potential” OR “laser evoked potentials” OR “nerve growth factor”) NEAR/5 pain) OR (electric* NEAR/1 pain*) OR (electric* NEAR/1 stimul*) OR (mechanic* NEAR/1 pain*) OR (mechanic* NEAR/1 stimul*) OR (thermal* NEAR/1 pain*) OR (thermal* NEAR/1 stimul*) OR (chemical* NEAR/1 pain*) OR (chemical* NEAR/1 stimul*) OR (cutaneous NEAR/1 pain*) OR (cutaneous NEAR/1 stimul*))

AND

TI/AB = (“back pain” OR “back ache” OR backache* OR “low back pain” OR “lower back pain” OR lumbago OR LBP OR “lumbar pain” OR “lumbar spine” OR “low back” OR “lower back”)
